# Generating randomness: making the most out of disordering a false order into a real one

**DOI:** 10.1186/s12967-019-1798-2

**Published:** 2019-02-18

**Authors:** Yaron Ilan

**Affiliations:** Department of Medicine, Hadassah-Hebrew University Medical Center, Ein-Kerem, POB 1200, 91120 Jerusalem, Israel

**Keywords:** Randomness, Quantum physics, Random-number generators

## Abstract

Randomness is far from a disturbing disorder in nature. Rather, it underlies many processes and functions. Randomness can be used to improve the efficacy of development and of systems under certain conditions. Moreover, valid unpredictable random-number generators are needed for secure communication, rendering predictable pseudorandom strings unsuitable. This paper reviews methods of generating randomness in various fields. The potential use of these methods is also discussed. It is suggested that by disordering a “false order,” an effective disorder can be generated to improve the function of systems.

## Introduction

Randomness underlies many processes in nature. In terms of scientific investigations, randomness pertains to quantum mechanics, chemistry, and biological systems. Moreover, randomness can be used under some conditions to improve the function and efficacy of systems. For instance, noise can induce phenomena that cannot be understood from underlying deterministic models alone. Indeed, not all noise is similar, and each aspect of stochasticity can lead to new behavior [1]. Many systems, including communication systems, rely on random-number generators (RNGs) for encryption. True random number generators (TRNGs) are systems whose outputs cannot be determined, even if their internal structure and response history are known. By contrast, pseudorandom number generators (PRNGs) produce sequences of numbers that are completely predictable though not easily distinguished from sequences obtained by truly random methods. Security can be established only if an RNG satisfies two conditions. First, the user must know how numbers have been generated to verify the validity of a procedure. This is unrealistic because RNGs can deviate from their intended plan owing to imperfections, component ageing, failures, or explicit tampering. The second requirement is that the system must be a black box from an adversary’s perspective. However, RNGs violate Kerckhoffs’s principle insofar as “the enemy knows the system being used” [2]. Thus cryptographic systems should be designed under the assumption that adversaries are familiar with them [2]. Quantum random generators (QRNGs) can overcome some of these obstacles. In what follows, we review methods of generating randomness in various systems and their potential use.

### Generating and using randomness in physics and algorithm development

Random-number generators require high-quality sources of random numbers, yet effective methods are needed for assessing whether a source produces truly random sequences. The sole demonstrations of TRNGs have proceeded through thermal noise and/or quantum effects, and this approach is expensive and requires complex equipment. Current methods either do not rely on a formal description of randomness (e.g., the NIST test suite) or are inapplicable in principle, requiring testing of all possible computer programs that could produce the sequence. A method that behaves like a genuine QRNG and overcomes these difficulties based on Bayesian model selection was proposed [3]. Moreover, hardware TRNGs are used to create encryption keys, and offer advantages over software PRNGs. However, the majority of devices and sensors require small, low-cost, and mechanically flexible TRNGs with low computational complexity. These rigorous constraints position solution-processed semiconducting single-walled carbon nanotubes as candidates. A TRNG that uses static random access memory cells based on solution-processed carbon nanotubes to digitize thermal noise was used to generate random bits [4]. The thermodynamic costs of the three main approaches to generating random numbers via the recently introduced Information Processing Second Law were presented [5]. Given access to a specified source of randomness, the RNG produces samples from a desired target probability distribution. This differs from PRNGs, which use wholly deterministic algorithms, and from TRNGs in which the source of randomness is a physical system. The thermodynamics of generators enables direct bounds on the required physical resources, specifically on heat dissipation and work consumption during the operation of several classes of RNG methods. TRNGs can generate random numbers and convert thermal energy to stored work [5]. A self-powered TRNG was proposed that uses triboelectric technology to collect random signals from nature [6]. It is based on coupled triboelectric and electrostatic induction effects at a liquid-dielectric interface that includes an elaborately designed triboelectric generator with an irregular grating structure, an electronic–optical device, and an optical–electronic device. The generator has nonlinear input–output behavior, contributing to increased randomness. Random number sequences are deduced from electrical signals received by an optical-electronic device.

Physical layer security uses the randomness of the wireless transmission channel for security. However, it is limited insofar as the main channel must be better than the eavesdropper channel. Cooperative communication was shown to ease this difficulty [7]. An actual-size concave grating with structural randomness was numerically analyzed. Numerical electromagnetic analyses that solve Maxwell’s equations provide diffracted fields and polarization characteristics. A difference-field boundary element method for analyzing actual-size concave gratings with 10,000 random blazed grooves was also proposed [8]. It offers vectorial diffracted or scattered waves with low computational resources. Relations between the degree of randomness and the diffraction efficiency were shown, including the polarization dependency. The path effect on a polished surface always matches tool motions, manifested as mid-spatial frequency errors in magnetorheological jet polishing processing. The Zernike extension and Neighbor–Gerchberg extension were used to design an extended surface with a weak edge effect in simulation [9]. Under the constraint of a pitch principle, a unicursal part-constrained path enhances the randomness of the tool path, including path turns and dwell-point positions. Linear canonical transforms (LCTs) are a family of integral transforms with wide applications in optical, acoustical, electromagnetic, and other wave propagation problems. The random discrete linear canonical transform (RDLCT) was designed by randomizing a kernel transform matrix of the discrete linear canonical transform (DLCT) [10]. It offers a greater degree of randomness because of the randomization of both eigenvectors and eigenvalues. The magnitude and phase of the RDLCT output are both random and can be used for image encryption. An entropy extraction mechanism based on sampling phase jitter in ring oscillators was designed to make high throughput TRNGs in a field programmable gate array [11]. A multi-phase sampling method was used to harvest the clock jitter with maximum entropy and fast sampling speeds. An evaluation of its randomness and its robustness to ambient temperature confirmed that this purely digital method provides high-speed high-quality random bit sequences. Ring oscillator-based physical unclonable functions protect the security of sensor nodes by generating random responses for a key extraction mechanism. They prevent non-volatile memory from storing secret keys. The hardware efficiency, randomness, uniqueness, and reliability of wireless sensor networks (WSNs) are also of relevance. A configurable device based on exclusive-or gates was used to increase hardware efficiency and mitigate vulnerability to EM analysis attacks, passing NIST’s statistical test [12].

A new type of elementary logic circuit, named random flip-flop, was also described [13]. Its action is maximally unpredictable and derives from a fundamentally random process of emission and detection of light quanta. It differs from conventional Boolean logic circuits whose action is deterministic and highly reproducible. Applications include randomness-preserving frequency division, random frequency synthesis, and RNGs. Most optical-chaos-based RNGs produce random bit sequences by offline post-processing with large optical components. A real-time hardware implementation of a fast physical RNG with a photonic integrated circuit and a field programmable gate array electronic board was presented [14]. An encryption key generated by two distant complex nonlinear units, forced into synchronization by a chaotic driver was also described [15]. The latter can be implemented on photonic, optoelectronic or electronic platforms, with reconfigurable key bitstream generation from chaotic signals. Although derived from a deterministic process, this bit series fulfills randomness conditions. A realistic protocol was proposed that amplifies the randomness of Santha-Vazirani sources and produces cryptographically secure random bits [16]. The protocol amplifies any such source that is not fully deterministic into a fully random source; it tolerates a constant noise rate and is robust to general no-signaling adversaries. A TRNG using the intrinsic variation of memristors as a natural source of entropy was presented [17]. Random bits were produced by cyclically switching a pair of tantalum oxide-based memristors and comparing their resistance values in the off state. Using an alternating read scheme in the designed TRNG circuit improved the bias of random numbers (i.e., making them less biased). A spatial network was modeled as a soft random geometric graph with two sources of randomness: nodes located randomly in space, and links formed independently between pairs of nodes with probability given by a specified function of their mutual distance. When randomness arises in the node positions and pairwise connections, for a given pair distance, the corresponding edge state is a random variable. The conditional entropy of an ensemble given the node location distribution for hard and probabilistic pair connection functions was studied. A connection function that generates maximum entropy was described [18]. Finally, a secured broadcast single-pixel imaging system using block-permutated Hadamard basis patterns for illumination was proposed [19]. The randomness in permutation operations facilitates system security, with satisfactory imaging quality and efficiency. This type of single-pixel imaging provides a solution to developing secured imaging systems for non-visible wavebands.

Analytical expressions have been provided for the near- and far-field diffraction of random Ronchi diffraction gratings where the slits of the grating are randomly displaced around their periodical positions [20]. The effect of randomness in the position of the slits of the grating decreased the contrast and even disappearance of the self-images for high near-field randomness. Diffracted orders, inherent to lensless endoscopy using coherent beam-combining and aperiodic multicore fibers (MCFs) with periodically arranged cores, reduce the field-of-view (FoV). Randomness in MCF core positions increase the FoV to the diffraction limit set by a single fiber core, while maintaining the experimental feasibility of the MCF. This system is appropriate for beam scanning imaging by applying a tilt to the proximal wavefront [21]. Digitally simulating the intrinsic randomness of broadband light passing through a spiral phase plate was used to generate partially coherent vortex beams with an arbitrary azimuthal index using only a spatial light modulator [22]. Anderson localization has been observed in matter waves, optical waves, and acoustic waves, but its effect can also be induced in metallic nonlinear nanoparticle arrays excited by a random electrically driving field. The dipole-induced nonlinearity results in the ballistic expansion of dipole intensity during evolution. The randomness of the external driving field suppresses such an expansion. By increasing the strength of randomness above a threshold, a localized pattern of dipole intensity can be generated in metallic nanoparticle arrays. The generated Anderson localization is highly confined, with its size limited to the scale of the incident wavelength. The data facilitate the manipulations of electromagnetic fields in the scale of the wavelength [23]. A 2D mixed state for the polarization of light is represented by a combination of a pure state and a fully random state. A Mueller matrix is represented by a convex combination of a pure component and three additional components whose randomness is scaled objectively. Such decomposition characterizes the polarimetric randomness of a system represented by a given Mueller matrix and provides criteria for optimally filtering noise in experimental polarimetry [24]. An anti-velocity jamming strategy was proposed to enhance the ability of pulse-Doppler radar to detect moving targets in the presence of translational and/or micro-motion velocity jamming generated by digital radio frequency memory repeat jammers. The strategy uses random-pulse initial phase pulses as its transmitted signal and derives memory jammers that are not adaptable to the randomness of the initial phase of the transmitted pulses in the pulse repetition interval domain. An entropy-based multi-channel processing scheme was used to extract the information of the received signal without assuming that true and false targets should be included within one coherent processing interval [25].

Datasets in astronomics, genomics, internet search logs, sensor networks, and social network feeds are often employed. Generating such data is viewed as a sampling process from a so-called big source of at least a few gigabytes. Previous approaches to big sources rely on statistical assumptions about the samples. A method that extracts almost-uniform random bits from big sources was shown [26]. A method of generating chaotic maps with expected dynamics was proposed using the inherent relation between the Lyapunov exponents of a Cat map and its associated Cat matrix, constructing a -dimensional (-D) hyperchaotic Cat map with any desired number of positive Lyapunov exponents [27]. The model constructs a -D hyperchaotic Cat map with less computation complexity and outputs with demonstrably strong randomness. Generating random bits from uncertain events whose outcomes are routinely recorded in the form of massive datasets has been studied. For instance, a PRNG that computes the chaotic true orbits of a Bernoulli map on quadratic algebraic integers was proposed [28]. It offers a mode for selecting the initial points (or seeds) to generate multiple pseudorandom binary sequences. It distributes the initial points almost uniformly in the unit interval, guaranteeing that the latter parts of the generated sequences do not coincide. A new PRNG was generated using a chaotic map of a dynamic parameter-control chaotic system [29]. This model of 1-D chaotic maps has a simple structure that uses outputs of a chaotic map (control map) to dynamically control the parameters of another chaotic map (seed map). The model produces many new chaotic maps that are sensitive to their initial states, and have wider chaotic ranges, better unpredictability, and more complex chaotic behavior than their seed maps. A method of composing new orbits from a given chaotic map was presented [30]. It tests discrete-time chaotic maps in a “deep-zoom” manner using k-digits to the right of the decimal separator of a given point from the underlying chaotic map. Rapid randomization was observed, whereby chaotic patterns became indistinguishable from the original orbits of the underlying chaotic map. Using this randomization improvement, a PRNG based on the k-logistic map was proposed.

Randomness is also used to improve modeling. Observer model performance was evaluated using data from GEANT4 Monte Carlo simulations for photons using custom models of plutonium inspection objects and a radiation imaging system [31]. The ideal observer was studied under signal-known-exactly conditions and in the presence of unknowns such as object orientation and absolute count-rate variability. When these additional sources of randomness were present, their incorporation into the observer yielded superior performance. Automatic Web-service selection is a research tool with which predictions of quality of service are possible based on historical service invocations [32]. As such, highly accurate predictions of missing quality-of-service data can be made by building an ensemble of non-negative latent factor models. These are diversified through feature sampling and randomness injection. Studies of randomized local binary features are used with methods such as Random Forests, Random Ferns, BRIEF, ORB, and AKAZE. With these methods, the randomness of operators reflects the sampling position. The quality of the binary feature space can be improved by increasing the randomness using a Randomized Intensity Difference (RID) operator to observe image patches. Compared to traditional incompletely randomized binary features (RIT features), randomized sampling generates a higher-quality binary feature space [33]. The phase diversity (PD) technique requires optimization algorithms to minimize the error metric and find the global minimum. Particle swarm optimization is suitable for PD due to its simple structure, fast convergence, and global searching ability. However, it suffers from a stagnation problem that can lead to incorrect solutions. To solve this problem, an inherent optimization mechanism was proposed. To improve the efficiency of this redistribution mechanism, randomized Halton sequences were introduced to ensure a uniform distribution and randomness of the redistributed particles in the search space [34]. Using a lightweight random partitioning scheme together with a carefully designed merging algorithm with results from random partitions overcomes the problem of scalable causal discovery used for biomedical studies and social network evolution [35].

### Generating and using quantum-based randomness

In many cases, using standard methods to generate randomness involves concepts that are impractical. Quantum-based randomness provides a means of generating genuine randomness that is impossible with classical deterministic processes. The unpredictability of randomness can be certified in a manner that is independent of implementation devices [36]. Intrinsic randomness is central to device-independent quantum technologies [37]. Two principles underlie the strain between quantum physics and local realism. The first is locality. Observing a particle at one physical location cannot have immediate effects on the properties of a particle at a different location. Indeed, no effect can travel faster than the speed of light. The second is realism, which expresses how the observable features of particles and photon polarizations exist, even if we do not actively measure them. Local realism means that two distant objects have only limited correlations: events undergone by one object cannot be correlated to another beyond a certain degree. Bell formulated this limit between physical objects in mathematical inequalities [38]. However, in quantum mechanics, correlations between distant particles exist, violating local realism. Events between quantum particles are indeed correlated, wherever they are in the universe. The hypothesis is that unknown physical parameters exist, such that the constraint imposed by inequalities would be correct all the same. It is thus possible to have two correlated particles that are distant from each other. By measuring the first we can learn something about the second without observing it directly [38].

Bell offered a way to tackle the threat to local realism posed by quantum mechanics: by studying quantum correlations in the form of entanglement [38]. Bell described local realism with a statistical limit such that if the results of an experiment violate Bell’s inequality, the local hidden-variable (LHV) model is not explanatory [39]. The Bell test examines whether or not the real world satisfies local realism, which requires the presence of some which are not a feature of quantum theory, to explain the behavior of particles like photons and electrons. If nature functions in accordance with any theory of LHVs, then the results of the test will be constrained in a particular, quantifiable way [39]. The Bell test assumes that no signal travels faster than light, and that it requires spatially distributed entanglement, fast and efficient detection, and unpredictable measurement settings [40–43]. Bell defined the LHV model as a class of non-quantum theories that are simultaneously local and realist [40]. Studies on device-independent quantum information demonstrate that Bell inequality violations (BIVs) challenge causal determinism [44]. This is a necessary and sufficient condition for the quantum protocol to overcome classical protocols [45]. A BIV can only be explained within local realism when events across history conspire to produce measured outcomes [46, 47]. Free variables are thus used to select measurements [48]. If some processes are “free” in the required sense, then other processes are similarly free [49]. This conditional relation leaves open the freedom-of-choice loophole, which defines the option that hidden variables influence setting choices. Such freedom is uncertain within local realism, and tests must assume physical indeterminacy [42]. Bell tests confirm the validity of quantum theory, but they leave open the option of non-quantum explanations as to why local realism is violated. Thus, physicists have been looking for ways to close these loopholes [38]. Many types of Bell tests have been proposed in an effort to do so [42]. BIVs have been observed in experiments that showed a qualitative connection to randomness. However, most experiments that violate Bell inequalities are nevertheless affected by loopholes, and cannot be considered black-box demonstrations [2].

Quantum randomness is based on BIVs. Such randomness is device-independent. That is, it does rely on any particular device model [44, 50]. The entanglement properties of random quantum states and dynamics are important [51]. Quantum randomness is the result of context and quantization. This approach challenges reductionist methods that seek to preserve classical physical theories [52]. QRNGs harness the intrinsic randomness in measurement processes. Their measurement outputs are truly random, given that the input state is a superposition of the eigenstates of the measurement operators [53]. QRNGs are ideal due to their intrinsic uncertainty [54]. Adversaries have no knowledge of their internal mechanisms, even though they have a full description of it [2]. However, the generation of pseudorandomness is much harder in the quantum case. Random quantum unitary time evolutions, or circuits, are a potent source of quantum pseudorandomness. They can sometimes replace fully random operations. Generic quantum dynamics cannot be distinguished from truly random processes [55]. There are many methods of generating quantum randomness in which the final random bit sequences pass all the NIST-STS and DIEHARD tests.

It follows from this that BIVs provide an experimental signature of randomness. BIVs can be verified by a user only from the statistics of the observed outputs of such processes. The verification procedure represents a black-box test of randomness [2, 56]. The limitation that any two photons must exchange signals at subluminal speeds was not enforced in the demonstrations of randomness generation based on Bell inequalities [4, 15]. An experiment was thus conducted with two photons in an entangled state such that their properties were strongly correlated. Each photon was sent to a different remote measurement station, where their polarizations were recorded. The photons were unable to interact given their distance, unless their signals travelled faster than the speed of light. Nonetheless, their measurement outcomes were correlated because of the photons’ entangled nature. The measurement outcomes were thus unpredictable, due to the strongly correlated behavior and distance of the photons. However, their randomness was small, even after millions of runs. A post-processing technique was used to generate truly random bits from these measurements, with minimal physical assumptions about the photons’ behavior [56]. Improved models were developed to explain the realization of such randomness [2, 56]. Over many runs, the sequence of measurement outcomes gathered enough uncertainty that truly random bits could be extracted. A method that weakened BIVs for generated random bits was developed as a secure QRNG [2].

Random Gaussian-free fermions satisfy the eigenstate thermalization hypothesis in the multiparticle sector, by analytically computing the correlations and entanglement entropies of the theory. The differences between fully random Hamiltonians and random Gaussian systems were described, providing a physically motivated notion of the randomness of a microscopic quantum state [57]. Electron transport through a nanoscale system is an inherently stochastic quantum mechanical process. Given that an electron has tunneled into an electronically unoccupied system from the source electrode at some particular time, the time it takes for it to tunnel out to the drain electrode is calculated [58]. Resonant tunneling diodes are thus used as practical true random number generators based on quantum mechanical effects [54]. A viable source of unbiased quantum random numbers was presented, the statistical properties of which can be arbitrarily programmed without the need for post-processing [59]. The method is based on measuring the arrival time of single photons in shaped temporal modes tailored with an electro-optical modulator. For a system of visual phototransduction, the process responsible for converting photons of light into usable electrical signals (or quantum bumps) requires randomness in both the photon inputs, regarded as extrinsic noise, and the conversion process, or intrinsic noise. Quantifying the relative effects of extrinsic and intrinsic noise has been studied. One such recent study in invertebrate phototransduction used minimum mean squared error reconstruction techniques based on Bayesian point process filters [60]. The algorithm estimates photon times from quantum bumps and uses Snyder filters to estimate random light intensities. The dominant noise source transitions from extrinsic to intrinsic as the light intensity increases with a delay that is critical insofar it can limit the speed at which invertebrates respond to stimuli.

Furthermore, true randomness can be generated from a mixed state if a system entangled with that mixed state is well protected. RNG based on measuring the quadrature fluctuations of a single-mode thermal state using an optical homodyne detector was demonstrated [61]. By mixing the output of a broadband amplified spontaneous emission source with a single-mode local oscillator at a beam splitter and performing differential photo-detection, the quadrature fluctuation of a single-mode output of the amplified spontaneous emission source was perceived. The model tolerated much higher detector noise than QRNGs based on measuring vacuum noise [53]. An all-optical QRNG using a dual-pumped degenerate optical parametric oscillator in a silicon nitride microresonator was developed. Quantum entanglement in magnetic materials produces a quantum spin liquid, in which strong quantum fluctuations prevent magnetic ordering even at zero temperature. A quantum spin liquid state was described in a spin-1/2 honeycomb lattice with randomness in the exchange interaction. Randomness was introduced into the organic radial-based complex leading to a random-singlet state. The magnetic and thermodynamic data supported liquid-like behavior consistent with that expected in the random-singlet state [62]. Polarization is a fundamental property of light. A dynamically unpolarized single-photon emission from a single [111]-oriented nitrogen-vacancy center in diamond was shown [63]. In this system the single-photon stream is unpolarized, exhibiting intrinsic randomness with vanishing polarization correlation between time-adjacent photons. It thus allows for true RNG. A practical random bit generation method was proposed based on the detections of a coherent state in the few-photon regime by a gated single-photon threshold detector, operating at the telecom wavelength of 1550 nm [64]. The method was applied in a free-running single-photon detector for increased throughput by chopping the light signal instead of gating the detector. A self-testing QRNG from a prepare-and-measure scenario with independent devices was proposed [65]. The Han16 protocol doubles the generation rate of the quantum random number compared with previous protocols. The protocol tolerates loss and noise. Device-independent QRNG based on a detection-loophole-free Bell test with entangled photons was also described [36].

A model for QRNG based on a random population of the output spatial modes of a beam splitter was shown when both inputs are simultaneously fed with indistinguishable weak coherent states [66]. Generating random bits as a function of the average photon number per input was demonstrated. Interference reduced the probability of coincident counts between the detectors associated with bits 0 and 1, increasing the probability of a valid output. A QRNG was described by measuring the amplified spontaneous emission noise of superluminescent light-emitting diodes [67]. By detecting and amplifying spontaneous emission noise, randomness extraction was integrated in a field programmable gate array. A method to extract randomness and achieve an entropy source for an RNG was described [68]. Its photon statistics and the bunching of a semiconductor laser with external optical feedback were studied. In a chaotic regime, the photon number underwent a transition from a Bose–Einstein distribution to a Poisson distribution. The second-order degree of coherence decreased gradually from 2 to 1. Based on a Hanbury Brown–Twiss scheme, pronounced photon bunching was noted for various injection currents and feedback strengths, suggesting randomness of the associated emission light. A high-speed physical random bit generator at gigabits per second without a time-delay signature was shown based on chaotic power fluctuations of a random fiber laser [69]. It was configured by means of a ring structure with semiconductor optical amplifiers as the optical gain and a fiber random grating as the random feedback medium. Its rate and randomness were limited by laser relaxation oscillation and external-cavity resonance and can be improved by post-processing. A chaotic external-cavity semiconductor laser is an entropy source for generating high-speed physical random bits. Physical broadband white chaos generated by optical heterodyning of two lasers was described as an entropy source to construct high-speed random bit generation with minimal post-processing [70]. Following quantization with a multi-bit analog–digital convertor, random bits were obtained by extracting several least-significant bits. White chaos was produced with a high entropy rate by single-bit quantization. A real-time QRNG was designed by measuring laser phase fluctuations to generate ultra-high-speed random numbers [71]. The speed limit of a practical QRNG depends on the restricted speed of randomness extraction. A method for closing the gap between fast randomness generation and slow post-processing was thus proposed. A secure key distribution scheme based on the dynamic chaos synchronization of two external cavity vertical-cavity surface-emitting lasers subject to symmetric random-polarization injections was demonstrated [72]. By exchanging random parameters that control the polarization angles of the driving injection, Alice and Bob identified the time slots in which high-quality private chaos synchronization was achieved, and independently generated a shared key from the synchronized polarization difference signals of their local lasers. Randomness generated by an optically injected semiconductor laser in chaos was studied by state-space reconstruction [73]. Randomness was evaluated by the divergence of neighboring states, quantified by time-dependent exponents (TDEs). The mean TDE is observed to be positive as it increases over time through chaotic mixing. At constant laser noise strength, the mean TDE for chaos was greater than that for periodic dynamics, attributed to the effect of noise amplification by chaos. After discretization, the Shannon entropies generated by the laser for the output bits were estimated to provide a fundamental basis for random bit generation. An ultra-fast physical RNG utilizing a photonic integrated device-based broadband chaotic source with a simple post-data processing method was also described [74]. The compact chaotic source is implemented using a monolithic integrated dual-mode amplified feedback laser with self-injection, where a robust chaotic signal with RF frequency coverage is generated. A real-time scheme for ultrafast random number (RN) extraction from a broadband photonic entropy source was proposed [75]. Ultralow jitter mode-locked pulses were used to sample the stochastic intensity fluctuations of the entropy source in the optical domain. Discrete self-delay comparison technology was used to quantize the sampled pulses into continuous RN streams. The model is bias free, eliminating the electronic jitter bottleneck confronted by currently available physical RN generators, and it has no need for threshold tuning and post-processing. Two strings of quantum random numbers simultaneously generated from the intensity fluctuations of twin beams generated by a nondegenerate optical parametric oscillator were proposed [76]. These were extracted with a post-processing algorithm by post-selecting identical data from two raw sequences and using a hash function.

Tests using physical randomness generators to choose measurement settings demonstrated a relationship between physical processes. If spontaneous emission is “free,” the outcomes of measurements on entangled electrons will also be free [49, 77]. One obstacle to manually done Bell tests is generating enough choices for statistically significance. A person can only generate three random bits per second, whereas a strong test requires millions of setting choices within minutes to hours. To achieve such rates, 100,000 human participants played an online video game that incentivized fast, sustained input of unpredictable selections and illustrated Bell-test methodology [78]. The game rewarded sustained, high-rate input of unpredictable bits. The participants’ choices were tested in various laboratories that verified local realism using photons [79, 80], single atoms [81], atomic ensembles [82] and superconducting devices [83]. The data confirmed the violation of Bell inequalities. Measurement-setting independence, provided by human agency, disagrees with causal determinism [44, 48]. The results thus closed the freedom-of-choice loophole—that setting choices are influenced by hidden variables to correlate with the properties of particles [84]. The human capacity for free choice eliminates the need for assumptions about physical indeterminism. Human choices show imperfect sequence randomness. Assuming no faster-than-light communication, such experiments prove that if human will is free, there are physical events that are intrinsically random, that is, impossible to predict [50].

### Generating and using randomness in chemistry

Randomness affects textural evolution. Plastically deformed metals are controlled by deterministic factors arising out of applied loads and by stochastic effects due to fluctuations of internal stress. Stochastic dislocation processes and inhomogeneous modes lead to randomness in the final deformation structure. Noise is involved in the analysis of a class of linear and nonlinear Wiener and Ornstein–Uhlenbeck processes. Linear Wiener processes are unaffected by the second time scale in the problem [85]. Silicon chips are vulnerable to counterfeiting, tampering and information leakage through side-channel attacks. However, an unclonable electronic random structure was constructed at low cost from carbon nanotubes [86]. This method uses two-dimensional random bit arrays to generate a ternary-bit architecture and a secure cryptographic key. A method for generating robust security primitives from layered transition metal dichalcogenides was described [87]. Physically unclonable primitives from layered molybdenum disulfide were designed by leveraging the natural randomness of their island growth during chemical vapor deposition. The distribution of islands on the film exhibits complete spatial randomness. The feasibility of embedding periodically arranged squares with a planar and vertical texture was demonstrated [88]. Using the natural randomness and uncontrollable variations of fingerprint textures, a polymer-stabilized graphic cholesteric liquid crystal symbol with a 2D barcode pattern was implemented with enhanced anti-counterfeiting features and improved security. Randomness was also important to the development of an electronic nose that distinguishes indoor pollutants [89]. The gas recognition rate was improved using an enhanced krill herd algorithm that relies on randomness to converge rapidly. Many attempts have been proposed to control crack formation, which compromises the strength and integrity of materials. A method to create modified films using electroplating on a pre-patterned substrate was used [90]. In thicker films, some randomness in the characteristic sizes of the fragments was introduced due to competition between crack propagation and crack creation. This method generated high-performance electrochromic structures. Surfactants provide an approach to building in randomness in generated magnetic behavior that can be manipulated via the formation of micelles and the design of a surfactant molecular architecture [91].

Activated carbon was synthesized with a chemical activation process [92]. Thermodynamic experiments suggested that the adsorption was spontaneous, endothermic, and increasingly random. Graphene oxide aerogels were used for adsorption of lead(II) ions from aqueous solutions [93]. The aerogels were fabricated from graphene oxide colloidal suspensions. Thermodynamic analysis demonstrated that its adsorption process was also spontaneous and endothermic with increased randomness at the solid–liquid interface. An activated carbon fiber/graphene oxide/polyethyleneimine composite was fabricated [94]. Its adsorption kinetics showed that the kinetic data fit with a pseudo-second-order kinetic model. Thermodynamic parameters showed that the adsorption process was spontaneous, endothermic and increasingly random. Similarly, a magnetic Schiff’s base chitosan composite was prepared whose sorption was endothermic, spontaneous, increasingly random [95]. Indeed, increased randomness was shown in several chemical reactions as a part of improving the function of the reactions. A chitosan-g-itaconic acid/bentonite and chitosan/bentonite nanocomposites were made for the adsorption of methylene blue from an aqueous solution [96]. The kinetic results indicated that the adsorption fitted with a pseudo-second-order kinetic model that suggested random adsorption at the interface. Polypyrrole wrapped oxidized multiwalled carbon nanotube nanocomposites were prepared via in situ chemical polymerization of pyrrole monomer in the presence of an oxidant [97]. The calculated values of the thermodynamic parameters showed that the adsorption process was spontaneous, endothermic, and marked with increased randomness at the solid–liquid interface. Solid waste from Jordanian olive oil processing was used to prepare biochar samples with increased randomness of the interface during the adsorption process [98]. Humic acid derived from rice straw was demonstrated to have Cu sorption that is endothermic, spontaneous, increasingly random [99]. The adsorption of chemical oxygen demand and biological oxygen demand from treated sewage with low-cost activated carbon was studied [100]. The results indicated that the adsorption was spontaneous, endothermic, and increasingly random. An activated carbon fiber modified by nitric acid was studied with absorption kinetics described by a pseudo-second-order model [101]. Again, the adsorption was shown to be spontaneous, endothermic, and increasingly random, as it was when studying the adsorption of copper ions onto chitosan films [102], a Gum xanthan-psyllium hybrid backbone graft co-polymerized with polyacrylic acid-co-polyitaconic acid chains [103], the removal of Chromium from an aqueous solution using sulfuric- and phosphoric-acid-activated Strychnine tree fruit shells as biosorbents [104], and the production of a polyvinyl alcohol–sodium alginate matrix embedded with red algae Jania rubens to remove lead from aqueous solutions [105].

### Randomness and human behavior

Random sequences have also been explored in psychology [106, 107]. For instance, humans do poorly when asked to produce a random sequence [106]. Furthermore, our choices are biased because when generating random sequences humans tend to systematically under- and over-represent certain subsequences relative to the number expected from an unbiased random process. Indeed, our choices contain statistical regularities, yet they also deviate from a uniform distribution [41]. For this reason, Bell argued that human choices could be considered free variables insofar as human intention and will is free [39]. Thus, experimental settings derived from human intentions fulfill the assumptions of Bell’s theorem [41]. In addition, common misperceptions of randomness reflect genuine aspects of the statistical environment. When cognitive constraints are taken into account they impact how that environment is experienced [108]. When people consider a series of random binary events, such as flipping a coin, they tend to erroneously underrate the probability of sequences with less internal structure [109]. This is explained by a so-called representativeness heuristic in which we assume that the properties of long sequences should also apply to those of short sequences. Imposing structure on randomness in the environment is evident in the gambler’s fallacy—the mistaken belief that, for example, after flipping a coin and getting Heads many times the occurrence of Tails is more likely [110]. A recent study showed that humans are susceptible to the bias of attributing more randomness to sequences with more alternation (e.g., when Heads follows Tails, rather than repeating). This so-called over-alternation bias was tested to determine its presence in stimuli that vary across feature dimensions, sensory modalities, presentation modes, and probing methods [111]. It was shown that participants judged over-alternating stimuli as the most random. This bias was consistent across temporal and spatial presentation modes, color and shape, sensory modalities, speed, stimulus size, and probing methods. The results suggested that the subjective concept of randomness is highly stable across stimulus variations.

The asymmetric measure of entropy has been suggested as an explanation for human biases and as a way to quantify subjective randomness [112]. A fitted asymmetric entropy measure was predictive when applied to different datasets of randomness-related tasks. Comparing human-generated sequences to unbiased process-generated binary sequences demonstrated that the constraints imposed on human experience provide a more meaningful picture of our ability to perceive randomness. A model of human random-sequence generation was thus proposed [108]. Binary sequences consist of a mixture of alternation and repetition. A study aimed to determine how people perceive such sequences and how they process alternation and repetition in binary sequences [113]. The data implied that, compared to repetition, alternation in a binary sequence is less noticeable. Human judgments were better explained by representativeness in the alternation rate than by objective probabilities. The data showed that participants were not sensitive to variation in the objective probabilities of a sub-sequence and used heuristics based on distinct forms of representativeness [109]. Research has also suggested that when generating random sequences, different participants adopt different cognitive strategies to suppress sequential dependencies [114].

### Applying randomness for overcoming tolerance to chronic therapies

Patients with drug-resistant epilepsy are particularly challenging to treat. Further, they often have poor prognoses in terms of seizure control, along with higher morbidity and mortality [115]. Drug resistance is a risk factor in status epilepticus and sudden death in epilepsy [116]. As the duration of the disease increases, there is a risk of drug resistance and polypharmacy. Worse, second-generation antiepileptic drugs provide no additional effect for poor responders to first-generation drugs [117]. Studies have proposed using randomness in such cases to partially overcome tolerance to therapies and to improve the efficacy of therapies [118–120].

In summary, randomness underlies many processes in various fields, and cannot be viewed as a disturbing disorder in nature. Randomness can indeed improve the efficacy of systems in physics, chemistry, biology, and psychology. Developing methods to better generate and understand randomness facilitates its use in these various fields.

## Conclusions

Randomness is far from a disturbing disorder in nature. Rather, it underlies many processes and functions. Randomness can be used to improve the efficacy of systems. Different methods of generating randomness are being explored in various fields. By disordering a “false order,” an effective disorder can be generated to improve the function of systems. Developing methods to better generate and understand randomness is expected to facilitate its use in these various fields.

## Abbreviations

RNGs: random-number generators
TRNGs: true random number generators
PRNGs: pseudorandom number generators
QRNGs: quantum random generators
LCTs: linear canonical transforms
RDLCT: random discrete linear canonical transform
DLCT: discrete linear canonical transform
NIST: National Institute of Standards and Technology
MCF: multicore fiber
FoV: field-of-view
RID: Randomized Intensity Difference
RIT: randomized binary features
PD: phase diversity
LHVs: local hidden variables
BIV: Bell inequality violation
TDEs: time-dependent exponents
RN: random number
